# Liquid Biopsy in Small Cell Lung Cancer—A Route to Improved Clinical Care?

**DOI:** 10.3390/cells9122586

**Published:** 2020-12-03

**Authors:** Matt Church, Louise Carter, Fiona Blackhall

**Affiliations:** 1 Division of Cancer Sciences, Faculty of Biology Medicine and Health, University of Manchester, Manchester M13 9PL, UK; matt.church@postgrad.manchester.ac.uk (M.C.); Louise.Carter24@nhs.net (L.C.); 2 The Christie NHS Foundation Trust, Wilmslow Road, Manchester M20 4BX, UK

**Keywords:** small-cell lung cancer, liquid biopsy, circulating tumour cells, circulating tumour DNA, cell-free DNA, personalised medicine, predictive biomarker

## Abstract

Small cell lung cancer (SCLC) has a particularly poor prognosis despite the high initial response to first-line systemic therapy, and there is a well-recognised lack of meaningful treatments beyond the second line. A number of reasons have been put forward to explain this, including a lack of common, easily-druggable genetic mutations in SCLC and rarity of high-quality tissue samples due to late presentation. Liquid biopsies, including circulating tumour cells (CTCs) and circulating tumour DNA (ctDNA) are increasingly used as surrogates for tumour tissue and have the advantage of being easily obtained serially to inform on the biology of disease progression and acquired chemoresistance, and may provide a pathway to improve care in this notoriously refractory disease. Here we discuss the current evidence behind these liquid biopsy methods in SCLC, and how they could be employed in future clinical care.

## 1. Introduction

Small cell lung cancer (SCLC) accounts for approximately 15% of all lung cancer cases [1] and is associated with a particularly poor prognosis despite significant initial response to first-line therapy [2]. While treatment is based on tumour, node, metastasis (TNM) staging, traditionally SCLC is broadly divided into limited stage disease which is confined to one hemithorax, and extensive stage disease with extra-thoracic spread. Untreated, survival is in the region of 1–3 months [3] and even with treatment, median survival is low at 15–20 months in limited stage and around 10 months in extensive stage [4,5].

While a minority of patients with limited stage disease have the option of surgery or primary radiotherapy, usually combined with chemotherapy, the first-line treatment for the majority has conventionally been a platinum-based doublet with etoposide or (in Asian ethnicities) irinotecan [5,6]. Recently, based on positive results from clinical trials, checkpoint inhibitor therapy has been added to this long-standing regime [7,8]. Treatment of four to six cycles of systemic drug therapies has response rates close to 70%, however, the almost universal relapse rate leads to the low survival outlined above [9]. Nonetheless, the high initial response to chemotherapy (with or without immunotherapy) often provides meaningful symptomatic benefit, and can therefore be used (sometimes as a single agent) in patients of higher performance status [10]. Patients are also divided into those who are “chemosensitive”, who do not progress in the first 90 days after the end of first-line therapy, and “chemoresistant” should they progress within the 90 days. Although controversial, prophylactic cranial irradiation (PCI) is offered in patients of good performance status who respond to chemotherapy [5]. In the second-line, chemosensitive patients are re-challenged with a platinum doublet and for chemoresistant patients, topotecan is recommended (although has limited survival benefit) [11]. There is no accepted third-line treatment [5].

While other cancer subtypes have seen huge shifts in treatment recommendations in line with modern breakthroughs in immune-oncology and targeted therapy treatments, the clinical care of patients with SCLC has, with the exception of the recent addition of checkpoint inhibitors into first-line therapy, remained the same for a number of decades [7,8]. Indeed, despite this change to SCLC treatment, questions still remain over which patients will most benefit from the addition of immunotherapy. Additionally, while many other forms of cancer are employing biomarkers for treatment selection, SCLC has lagged behind, partly due to genomic complexity and lack of routine tissue. Liquid biopsy may represent a method to “level up” SCLC in this regard. This review will assess the current role of liquid biopsy methods in SCLC and discuss the role these technologies could play in the next decades of SCLC clinical care.

## 2. Current Issues in Small Cell Lung Cancer

A number of reasons have been put forward to account for the lack of progress seen in SCLC compared to other cancer types, especially non-small cell lung cancers (NSCLCs). While the recent improvements seen in cancer survival come in part from earlier diagnosis of disease, another pillar of improvement has been increasingly biomarker-driven “personalised” or “targeted” treatments and immunotherapy [12]. Such a process relies on an ample supply of tumour material to perform biomarker and genetic analysis upon, which is often lacking in SCLC. While tissue is generally obtained through biopsy at diagnosis for histological confirmation, large resections from surgery are exceedingly rare [5] and biopsy samples, which are generally fine-needle aspiration, are not always of sufficient quality for biomarker analysis [13].

Indeed, the inherent late presentation and short doubling time seen in SCLC, alongside the high potential for a response, generally leads to chemotherapy being started rapidly after diagnosis, reducing the ability to wait on complex genetic analysis to be performed and interpreted. Arguably, the ideal time for biomarker directed treatment in SCLC would therefore be in the second-line or later. However, the use of diagnostic tissue analysis in consideration of later line therapy is limited by acquired chemoresistance and changes in clonal dominance generated during therapy. Serial tissue biopsies which would remove this obstacle are very rarely performed in SCLC. A further complication to the tissue analysis approach in SCLC is the degree of intratumoural heterogeneity seen in this disease, especially following chemotherapy [13,14].

**Figure 1:**
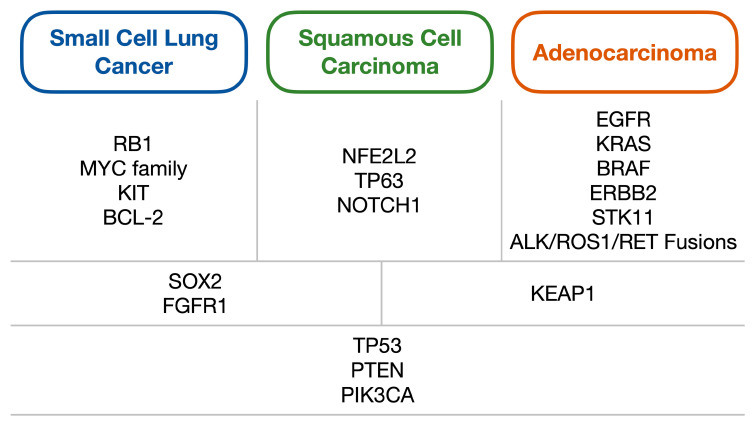
Diagram of the common genetic alterations found within lung carcinomas, split into alterations generally associated with either small cell lung cancer, squamous cell carcinoma or adenocarcinoma (first row); those commonly shared between two of the three subtypes (second row); and alterations shared between all three (third row). Adapted from Pietanza and Ladanyi (2012) [21].

SCLC is heavily associated with a history of smoking, and consequently incidence has been declining in the western world but appears to be increasing in Asia in line with changes in smoking prevalence globally [15,16]. Correspondingly, SCLC has a high mutational load similar to other tobacco-driven cancers, with one study finding a rate of 8.62 nonsynonymous mutations per million base pairs [17]. In regards to specific genomic alterations, *TP53* and *RB1* have been found to be near-universally inactivated [17], with other common alterations including changes in *MYC*, *BCL-2* and *KIT* [18]. More rarely, mutations in *PTEN*, *PIK3C, EP300* as well as *SOX2* and *FGFR1* amplifications have been reported [19,20]. As discussed by Pietanza and Ladanyi, the *SOX2* and *FGFR1* amplifications represent a genetic similarity to squamous cell carcinoma (which is also commonly smoking-related) (Figure 1) [21]. However, in comparison to NSCLC, aberrations in targetable oncogenes such as *EGFR* and *ALK* are rarely found [2], and indeed it is notable that many common mutations found in SCLC are tumour suppressor genes rather than oncogenes. This highlights a key issue with drug development in SCLC, given that tumour suppressor gene alterations are generally much more heterogeneous than oncogenes and are therefore more challenging to develop effective specific small molecular inhibitors against.

As discussed, SCLC has a particularly high mutational burden, and indeed it was initially proposed that this would render the disease especially sensitive to checkpoint-inhibition therapy as is seen in other cancer types [22]. However, despite recent successes with immunotherapy combined with first-line SCLC therapy, tumour molecular burden (TMB), which is a key predictive biomarker of immunotherapy benefit in other cancer types, has not been conclusively shown to be predictive in SCLC [23]. Similarly, while there is some evidence of programmed death-ligand 1 (PD-L1) status being a predictive biomarker for pembrolizumab’s response in SCLC, there have been contradictory reports for other checkpoint inhibitors [24].

To date, no biomarkers are routinely employed for treatment decision making in patients with SCLC. Two early proposed biomarkers for this condition were neuron-specific enolase (NSE) and pro-gastrin releasing peptide (pro-GRP). NSE is the most widely studied and has been found to be related to diagnosis and treatment response. NSE is raised in most SCLC cases, although to a larger degree in extensive stage disease. Levels appear to briefly increase after chemotherapy, before rapidly declining. This decline has been shown to correspond to radiological tumour response [25]. Both NSE and Pro-GRP, although to a greater extent NSE, have been found to be prognostic, with a 2001 study finding NSE ≥ 7.5 ng/mL infers poor prognosis (median survival 10.5 vs. 21.3 months) [26].

A recent attempt to develop a biomarker-driven treatment for SCLC was that of rovalpitumab tesirine, an antibody drug conjugate targeting the NOTCH ligand delta-like 3 protein (DLL-3). DLL-3 is expressed highly in SCLC, and while selection for DLL-3-high patients has shown some evidence of tumour response in the third-line, a large randomised phase III study was recently halted owing to poorer survival in the rovalpitumab tesirine arm compared to the standard of care, topotecan arm [27,28]. Another example was in a phase I trial of sonidegib, a hedgehog inhibitor, that showed an interesting report of a SOX2 amplification-positive patient being progression-free after 27 months of maintenance therapy, but this case report has yet to be validated as a true predictive biomarker [29].

As mentioned, alterations in the *MYC* family of genes are commonly found in SCLC. An ongoing randomised phase II trial of paclitaxel plus the aurora A kinase inhibitor alisertib or placebo in the second-line setting has shown favourable progression-free survival (PFS) benefits. Subgroup analysis published this year has shown a significant benefit in patients with *c-Myc* expression in the alisertib plus paclitaxel arm, highlighting another potential biomarker [30,31]. Similarly, in a study of temozolomide plus the PARP inhibitor veliparib (or placebo), while no difference in PFS or overall survival (OS) was seen between arms overall, a significant benefit in both PFS and OS was seen in the PARP inhibitor arm when split by immunohistochemical expression of SLFN11 [32]. While these studies do provide evidence to support a biomarker-driven approach in SCLC, unfortunately, these results remain remote for use within the clinic.

Another issue seen within SCLC is that of the significant mortality and rapid progression inherent to this aggressive disease. Indeed, one study has shown that 7.8% of patients with SCLC in the United Kingdom died within 30 days of chemotherapy administration [10]. As expected, 30-day mortality was associated with worse performance status and extensive stage disease. Even in those patients who do survive beyond 30 days, a subset will not respond to therapy and by the time of repeat radiological imaging to confirm this, may have clinically deteriorated to the point that further therapy is not recommended [33,34].

The rapid decline in a high proportion of SCLC patients who were deemed fit for chemotherapy at the point of commencing it, and the significant 30-days post-chemotherapy mortality, highlights an unmet need for methods to select patients who will or will not benefit from therapy. In addition, this further demonstrates the need for next-generation treatments, as these selection methods will be most advantageous if the number of effective treatment options in SCLC could be increased through preclinical and clinical trial research. In summary, there are a number of ongoing challenges in SCLC which liquid biopsy may help to address in the pursuit of improving clinical care (Table 1).

## 3. Liquid Biopsies in Small Cell Lung Cancer

Liquid biopsy, a term first used by Pantel and Alix-Panabières in 2010 to describe an investigation taken from the blood as a proxy to tissue sampling, has been studied in various settings for application in SCLC. Liquid biopsy has been investigated as both a preclinical tool to interrogate disease biology and to identify targets to exploit for therapeutic control, and in the clinical setting as a prognostic, predictive and pharmacodynamic biomarker or for the generation of tumour xenografts [35,36]. In contrast to other cancer subtypes, the majority of liquid biopsy research within SCLC has involved circulating tumour cells (CTCs), despite circulating tumour DNA (ctDNA) being generally considered the more “mature” liquid biopsy technology.

### 3.1. Circulating Tumour Cells in Small Cell Lung Cancer

**Figure 2:**
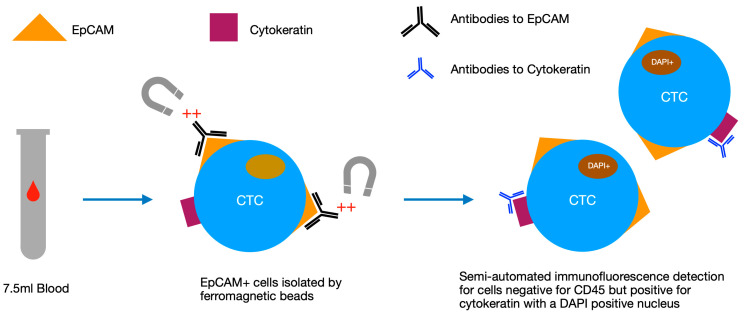
Diagram of the stages of analysis in the CellSearch^®^ system, which uses a semi-automated process to select for epithelial cells with specific antibody signatures and morphology. CTC: circulating tumour cell, EpCAM: epithelial cell adhesion, DAPI: 4′6-diamidino-2-phenylindole.

CTCs have been known to exist in the blood of cancer patients since the 19th century when they were first described by Ashworth [37]. Little research was carried out on these cells initially due to their comparative rarity in the blood, however, in the last few decades, advanced methods of isolation have permitted more detailed analysis and elucidation of CTCs. One of the more popular detection methods is the CellSearch^®^ system (Menarini-Silicon Biosystems). In this system, a 7.5 mL blood sample is enriched for epithelial cells using ferromagnetic beads coated in epithelial cell adhesion (EpCAM) antibodies, before then removing non-cancerous cells (including white blood cells) by detecting for oval-shaped cells positive for cytokeratin, containing a DAPI positive nucleus but negative for CD45 (Figure 2) [38]. Validation studies have shown that CTCs detected via this method are rare in healthy controls and vary depending on the cancer type and stage [38]. The CellSearch^®^ system is among the most commonly used CTC detection technologies, however other systems have been developed including CTC-iChip, EPISPOT and ISET among others [39,40,41,42]. Of these, ISET benefits from being less specific for epithelial-derived cells and therefore can isolate non-epithelial circulating cells [43].

Early results from this technology revealed that the number of CTCs per 7.5 mL of blood is prognostic in breast, prostate and colorectal cancer [44,45,46] and led to FDA approvals for use in these cancer types. A similar relationship has also been found in NSCLC, where baseline levels of CTCs < 5 or ≥ 5 divided the cohort into different prognostic groups. Those with ≥ 5 CTCs per 7.5 mL of blood at baseline had a PFS of 2.4 months and an OS of 4.3 months. Comparatively, those in the < 5 CTC group had a PFS of 6.8 months and an OS of 8.1 months [47].

In comparison to other cancer types, SCLC has been found to have significantly higher levels of CTC counts, with up to tens of thousands of CTCs found per 7.5 mL of blood [48,49]. Furthermore, a significant proportion of SCLC patients have CTCs, with most studies finding over two-thirds of examined patients to have in excess of two CTCs per 7.5 mL of blood. Limited stage patients generally have much lower CTC counts [50]. This high frequency and level of CTC burden is perhaps to be expected given the aggressive and highly metastatic nature of SCLC and its epithelial as well as neuroendocrine origin. Given that the rarity of CTCs is the main challenge found in CTC analysis, the natural suggestion has been that SCLC may be an ideal arena for CTC analysis and clinical utility.

Multiple studies over the last decade have shown the prognostic role of CTCs in SCLC (Table 2). Hou and colleagues were the first to use the CellSearch^®^ system in SCLC and showed in 2009 that CTCs could be found in 86% of patients at levels ranging from 0–44,896 per 7.5 mL of blood, with numbers correlated to liver metastasis, high alkaline phosphatase and low serum sodium. Baseline CTC count, when split into the highest and lowest quartile, showed a significant prognostic effect, with median survival 134 vs. 443 days respectively [51]. In 2012 Hou et al. found that a level of 50 CTCs was able to divide the cohort into “favourable” and “unfavourable” groups. OS and PFS was significantly reduced in those with ≥50 CTCs at baseline (OS 5.4 vs. 11.5 months, *p* < 0.0001). In addition to baseline levels, the number of CTCs following the first cycle of chemotherapy was also prognostic, as was change in CTC category; those with < 50 at both baseline and post cycle one having improved survival compared to those who converted from <50 to ≥50 or remained ≥50 at both timepoints [49].

The same year, Naito published similar results showing baseline levels to be prognostic, although in their analysis a cut-off of eight CTCs was selected. In comparison to Hou, in this study repeat CTCs were taken three weeks following completion of the last chemotherapy cycle or thoracic radiotherapy and at relapse. The results also showed the presence of ≥8 CTCs post-treatment and at relapse to be predictive of poor outcome (≥8 CTCs post-treatment giving post-treatment survival 4.1 months vs. 13.9 months for <8, *p* = 0.0096), although given the timing of collection this data is more relevant for subsequent lines of therapy [48]. Hiltermann used a similar methodology to Hou, showing that while baseline CTC below the lowest quartile (<2) was strongly prognostic, the numbers of CTCs after one cycle of chemotherapy was the strongest predictor of overall survival. CTC number after treatment completion was also related to survival, as in Naito’s data [50]. These findings are both significant clinically, as the remaining CTCs after one cycle or full course of first-line chemotherapy are representative of the resistant cells and are therefore relevant for subsequent treatment selection.

Notably, in both Hiltermann’s and Naito’s studies, no relationship was found between CTC levels and radiological response. Indeed, similar research in China has also shown no relationship between CTCs and radiological response [52]. However, it is clear from these studies that exposure to chemotherapy generally results in a significant pharmacodynamic fall in CTC counts from baseline. More recently, Aggarwal et al. published a series of 50 SCLC patients who had serial CTC sampling performed even within the first cycle. Patients where there was a fall in CTC level from baseline on day one, two or three of cycle one had longer OS compared to those who did not, although this was not statistically significant. This is of particular interest, as it proposes an option to re-evaluate the appropriateness of therapy within one cycle. It may be that those who do not see a rapid fall in CTCs would benefit from a change, or withdrawal, of therapy in light of possible chemotherapy adverse effects. Again, a decrease in CTCs was not correlated with radiological response [53].

In this same study, while a level of 50 CTCs was prognostic for both OS and PFS when a level of five CTCs was used, only PFS was significant at baseline. For post one cycle of chemotherapy, both levels were prognostic. Interestingly, the study measured the DNA damage marker phosphorylated H2AX (γH2AX) on CTCs, and demonstrated that patients with an increase in γH2AX-positive CTCs after chemotherapy had a longer OS (25.3 vs. 9 months), although (potentially due to low sample size) this was not statistically significant [53].

The fact that CTC number at baseline is prognostic for overall and progression-free survival has broad applicability in the clinic, both in providing clinicians with information to aid in their treatment decision and providing patients with sufficient knowledge to consent to therapy in what may be their last months of life. Indeed, a meta-analysis has confirmed the relationship of baseline CTC count to OS and PFS in SCLC [54]. It should be noted that the studies above generally include both extensive stage and limited stage patients in the same analysis, despite the significantly different prognosis and treatment strategies employed for each stage. A study specifically within extensive stage SCLC was performed by Normanno et al. and was designed to evaluate whether CTC number provides a clinically meaningful benefit to previously known prognostic effects (such as performance status, stage, and lactate dehydrogenase). Baseline numbers of CTCs added little to existing prognostic information in their model, however, a reduction in CTC level of 89% following one cycle of chemotherapy did significantly improve the prognostic accuracy of the model [55].

Given that one-third of SCLC patients present as limited stage [1], it is also of interest to evaluate the prognostic potential of CTCs within this group. This was recently performed as an exploratory analysis of the CONVERT randomised phase III trial comparing twice and once daily concurrent chemoradiotherapy. Of the patients analysed, 60% had CTCs, and using cut-off levels of 2, 15 and 50 were all prognostic for both PFS and OS [56]. The optimal prognostic level was found to be 15 CTCs, with OS of 5.9 months for ≥15 CTCs vs. 26.7 months for <15 CTCs. All patients with CTCs ≥15 found at baseline progressed and had died within two years. The authors propose that the discovery of high CTC numbers in limited stage disease implies aggressive biology and potential for further metastatic spread, and therefore may indicate a need for a post-primary treatment maintenance therapy should a suitable intervention (perhaps checkpoint inhibition), be shown to be valuable as maintenance in SCLC. It has also been proposed that CTCs could be used to increase the role of surgery in SCLC by selecting a group of patients with low CTC numbers (which implies a low risk of disseminated disease) for consideration of surgical management [57].

While the above studies have focused on the standard of care platinum-based chemotherapy or chemoradiotherapy, CTCs have also been investigated in the clinical trial setting. In one phase II trial of the multi-target tyrosine kinase inhibitor (TKI) pazopanib in the second-line setting, administration of pazopanib resulted in a significant decrease in median CTC numbers [58]. As is seen in the first-line, CTC level at baseline ≥5 was associated with a significantly shorter PFS (1.9 vs. 3.6 months, *p* < 0.001) and OS (5.2 vs. 10.1 months, *p* = 0.001). The trial also found a relationship between survival and number of CTCs post one cycle of therapy using multivariate analysis. A further analysis performed immunofluorescence staining on CTCs for the presence of VEGFR2, given that pazopanib is expected to inhibit VEGFR2. VEGFR2-positive CTCs decreased on treatment, but increased at progression, highlighting a potential mechanism for resistance to therapy. As another example, in the previously mentioned phase I trial of the hedgehog inhibitor sonidegib, the number of CTCs after one cycle was found to correspond to both overall survival and be predictive of response [29].

As is clear from the above, there is currently no agreed prognostic CTC count cut-off to use for clinical decision making, which limits the ability to translate these findings into the clinic. Despite this, given the clear and consistent relationships to survival seen in such studies, CTCs may yet have a significant role in clinical SCLC treatment selection going forward. In practice, this could involve patients with high CTC counts being directed to either more intense treatments, maintenance therapy, or avoiding chemotherapy if risks versus benefit are deemed too high for the individual patient. In addition, the failure to trigger a fall in CTCs during first-line therapy could prompt an early change in treatment strategy to improve outcomes. Prospective studies to test such hypotheses are currently lacking.

### 3.2. Circulating Tumour Cells for Genetic Analysis

**Figure 3:**
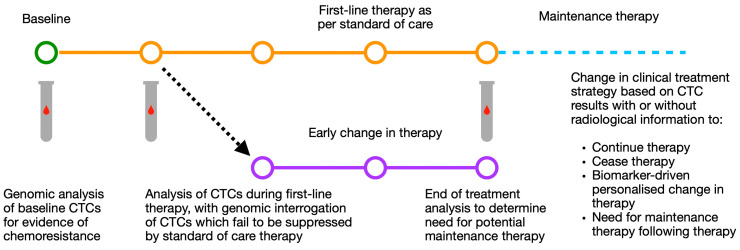
Schematic of possible routes for CTC based modification of individualised treatment strategy using baseline and mid-treatment CTC results by number and genomic analysis. CTC: circulating tumour cell.

While the change of treatment based on CTC numbers in the blood may be of benefit, it would be of greater value to use these CTCs to evaluate specifically where future treatment should be directed. Indeed, if baseline CTCs could provide possible individualised treatment options, or if CTCs which failed to respond to first-line treatment (and therefore represent the subclone of disease responsible for treatment failure) could be interrogated for biomarkers or treatment efficacy, this could have a meaningful impact on clinical care (Figure 3).

There is evidence that such an approach is feasible. CTCs, derived from the main mass of tumour, are thought to be excellent surrogates of main cancer bulk. One current issue in SCLC is the paucity of tumour tissue for comprehensive genomic analysis owing to low rates of surgical resection and poor biopsy material [13]. Genomic analysis of tumours is increasingly common in other cancer subtypes, especially NSCLC, and is fundamental to the view of cancer precision medicine. CTCs, as a surrogate of SCLC tissue, provide an opportunity to perform genomic interrogation of disease despite these inherent issues in SCLC.

The CellSearch^®^ system has been used to enrich CTCs from patients and then permit for CTCs to be isolated using DEPArray (Menarini-Silicon Biosystems). Genomic analysis of these isolated cells has shown common genetic changes in concordance with other SCLC genomic analyses [60]. In one study using this method, genome-wide copy-number aberrations (CNA) were analysed and patterns of copy number gain and loss showed broad concordance with previous studies. This data was subsequently used in a training set to generate a CNA signature that could predict chemosensitive or chemorefractory patients. In theory, such a signature could classify chemosensitivity at the start of treatment and provide a pathway for biomarker directed care. In serial CTC samples obtained following the chemosensitive patients’ eventual progression, there was little change in CNA, indicating that the de novo resistance mechanisms are inherently different to the acquired chemorefractory molecular mechanisms [60]. These findings not only highlight that genomic analysis is feasible from CTCs in SCLC, but also that it has a potential clinical application.

Other studies have also looked at the genomic information available from CTCs. In a Chinese study, single-cell sequencing of CTCs was performed on 48 patients using whole genome sequencing (WGS). Ten of these patients had matched tissue samples which were analysed in parallel, including patients where both primary tumour and metastatic lesion samples were obtained. It was found that 82% of mutations were shared between the primary tissue, metastatic tissue and the CTCs, and while there were some mutations unique to metastatic sites, this is to be expected given the knowledge of intratumoural heterogeneity and evolution of tumour subclones. Overall, the CTC-derived genomic information was highly representative [61]. A CNA scoring system was also prognostic for PFS and OS in this paper, further underling a potential clinical role for genomic information generated from CTCs.

### 3.3. Circulating Tumour Cells for Patient-Derived Cell and Animal Models

CTCs are postulated to be involved in metastatic spread, and therefore their tumorigenicity has been suggested for some time [42]. Indeed, recently this quality of CTCs has been applied to develop CTC-derived explants (CDx) in immune-compromised mice [62]. Hodgkinson and colleagues used this technique in 2014, by injecting CTCs from six chemotherapy-naïve extensive stage SCLC patients and showing the success of- and time to- palpable tumours relate to a number of CTCs (only samples with CTCs ≥ 400 per 7.5 mL were successful). Genomic and histological analysis showed that the CDx tumours retained SCLC characteristics from corresponding patients. Crucially, CDx models were then treated with either cisplatin and etoposide or vehicle, and responses were seen to mirror patient response and differences in overall survival [62]. Therefore, such models (which can be created within months) can be assessed for response to various therapies. Due to the rapid progression inherent to SCLC, this may not benefit first-line treatment, but CTCs obtained after the first cycle of first-line therapy could potentially be used to form CDx models for determining optimal second-line therapy as the first-line completes.

A similar study by Drapkin generated both traditionally derived patient-derived explant (PDx) models (from tissue biopsy material) as well as CTC-derived CDx models. Serial CDx models were developed from a single patient enrolled on a trial of olaparib and temozolomide post-first-line therapy. Models were generated at baseline and at time of relapse, with baseline models also responding dramatically to olaparib and temozolomide but progression samples not responding to the same [63]. CDx models have also been used to evaluate intratumoural heterogeneity following treatment resistance [13]. While these studies underscore the potential use of such models, factors such as the high concentration of CTCs required, laboratory availability and costs will likely present a barrier to clinical use and may result in this technique being limited to clinical trials.

To avoid the technical difficulties, long timelines and costs of in vivo CDx models, CTC-derived cultures have been suggested as an alternative. Hamilton and colleagues have used CTCs from SCLC patients to create CTC cultures specifically to assess the benefit of second-line therapy [64]. These CTC cultures were more chemosensitive to epirubicin compared to topotecan but also were more sensitive to these chemotherapies than traditional SCLC cells lines (derived from metastases), indicating that CTC reduction in patients on second-line therapy may overestimate chemotherapy effect on solid metastases. Other groups have also developed cultures from SCLC CTCs [63,65]. The results by Hamilton are particularly relevant as they focus on the second-line and given the much lower response rate to second-line therapy, there is scope to apply CTCs in this later stage setting. The results also suggest that while CTCs in culture can be useful, they should be used with caution, and indicate that CDx models may be more representative overall for augmenting clinical care.

### 3.4. Circulating Tumour DNA in Small Cell Lung Cancer

The high rates of CTCs in SCLC in comparison to other cancer types has led to a great deal of interest in CTCs as a liquid biopsy in SCLC. However, in other cancer types, it is generally accepted that circulating tumour DNA (ctDNA), which is a subset of total cell-free DNA (cfDNA), is the more mature liquid biopsy technology. Cancer patients have been known have comparatively higher concentrations of cfDNA since the 20th century [66], but the technology to accurately analyse this genomic fraction has only more recently become widely available. ctDNA is made up from both single and double-stranded DNA, and has a short half-life in the region of 2 h, highlighting its “real-time” relationship to tumour biology [67]. ctDNA is thought to be released through a combination of active cellular processes and neoplastic cell apoptosis. Indeed, ctDNA levels generally correspond to the overall tumour burden and stage [68,69]. SCLC, with its late presentation and widely disseminated disease, is, therefore, an ideal candidate for ctDNA analysis.

As with CTCs, the main obstacle in the analysis of ctDNA is its relative rarity within the blood, a problem amplified by lysis of white blood cells releasing non-cancerous cfDNA and diluting the ctDNA of interest [70]. Indeed, the lysis of white blood cells requires that ctDNA is analysed either within six hours post venesection from a standard EDTA bottle, or is collected into specialised stabilisation tubes to permit transport. As an alternative, samples can also be centrifuged locally and the plasma fraction transported frozen to the analytical laboratory [70].

At the processing lab, plasma is analysed for ctDNA by one of two broad methods. The first uses the polymerase chain reaction (PCR), which is widely used due to its simplicity and low cost. The sensitivity of these approaches has been greatly increased by new developments such as digital droplet PCR or BEAMing (beads, emulsions, amplification and magnetics). Unfortunately, these PCR based methods can only analyse for a small number of gene mutations known a priori, and therefore an arguably more powerful method of analysis is next-generation sequencing (NGS) of ctDNA. NGS can detect previously unknown genetic alterations as well as deletions, amplifications, rearrangements and translocations [71]. ctDNA analysis by both of these methods have shown high concordance with tissue analysis in a variety of tumour types [72,73,74].

One of the first published analyses of ctDNA in SCLC was in 2008, when cfDNA was compared between SCLC patients and healthy controls. The concentration of cfDNA was significantly higher in SCLC patients plasma than healthy controls and fragment sizes were also longer in SCLC samples [75]. Indeed, the observed differences between healthy controls and SCLC patients have suggested that ctDNA could be used to assist in the early detection of this poor-prognosis, late presentation disease in order to improve overall outcome. This possibility has been researched, with the inactivation of *TP53* (which is near-universal in SCLC) as a target. *TP53* mutations in the blood were detected in 49% of SCLC patients (35.7% stage I–II, 54.1% stage III–IV), but were also detected in 11.1% of non-cancer controls [76]. Such a high rate of *TP53* mutations in controls greatly restricts the potential of this method for early detection, especially given the high threshold of utility that must be demonstrated when developing an effective screening programme.

However, the main promise of ctDNA as a liquid biopsy is that of obtaining genomic information for use as a biomarker either at baseline or in tracking progress during treatment and relapse through serial biopsies. The feasibility of this process in SCLC has been demonstrated, with one study by Almodovar using an NGS approach to analyse 140 samples from 27 patients at various points through therapy. Results showed 85% of patients had disease-associated mutations detected in blood, at allele frequencies (AF) between 0.1 to 85% [34]. The most common mutations were in *TP53* and *RB1*, but others such as *MYC*, *MTCL1*, *PTEN*, *NOTCH1-4*, *KIT*, *BRAF* and *PIK3CA* were also found.

Longitudinal sampling of patients in this study generally showed a reduction in AF of baseline ctDNA mutations during therapy, with increases following the completion of chemotherapy but proceeding radiological progression. Similar results were observed during second-line treatment, for example a patient started on paclitaxel for second-line therapy was found to have pre-treatment *TP53* and *NOTCH3* mutations in ctDNA at AF of 6.7% and 2.4%, respectively. Within weeks of paclitaxel therapy, these mutations became undetectable. Six months after initiation of paclitaxel, the same *TP53* and *NOTCH3* mutations were detected (AF of 3.9% and 3.7%), although radiological imaging showed an ongoing response. However at the next sampling, AF of these mutations had increased over ten-fold and imaging showed widespread progression, highlighting that ctDNA liquid biopsy was able to detect early signs of recurrence in advance of traditional radiology [34].

In addition to showing response and later recurrence, this study was able to show early signs of resistance to therapy in some (later termed chemoresistant) patients. Given the potential adverse events associated with aggressive chemotherapy regimes in SCLC, early information on resistance may enable withdrawal of futile, possibly harmful, chemotherapy and improve the quality of life of these patients. Similarly, the authors suggest that clearance of ctDNA, alongside good response on imaging, could be used to avoid PCI in patients, again preventing unnecessary treatments and adverse effects. Finally, as is seen with number of CTCs, increased cfDNA (measured as “genomic equivalents”) was found to have a worse overall survival [34]. Other studies, also similar to results in CTCs, have shown that specific mutational profiles of SCLC ctDNA are associated with chemosensitivity to platinum treatment, which certainly has great relevance to clinical care and understanding of the mechanisms of chemoresistance [60,77]. Indeed certain mutations, such as *NOTCH1*, have been found to be enriched in post-treatment samples of ctDNA, indicating a potential role in chemoresistance and clonal evolution [78].

Another study compared ctDNA from SCLC patients with germline DNA from the same participant, and found a range of common mutations known in SCLC but also rarer mutations such as *ATM.* Survival analysis of mutations showed that mutations in *SETBP1* and *PBRM1* were associated with worse OS and PFS compared to wild-type, mutations in *ATM* were associated with poor PFS only and *ATRAX* and *EP300* alterations were associated with poor OS only [79]. A “mutation risk index” was developed and this was significantly related to both OS and PFS. Interestingly, more CNV changes and mutations were found in the cfDNA of SCLC than had been seen in the same group’s other studies of different tumour types, perhaps due to the high-doubling rate and high cell death in SCLC.

In the largest study to date, Devarakonda and colleagues collected samples from 564 SCLC patients at diagnosis or relapse and found 90% of patients had at least one genomic alteration [80]. As expected, mutations in *TP53* and *RB1* were most frequent, with a range of other alterations seen. It was noted that a higher percentage of alterations in *APC* and *AR* were found in relapse samples, suggesting a possible role in relapse and a potential molecular target in *AR*. Indeed, the authors looked in particular for potentially targetable alterations, finding *MYC* amplifications in 5% of diagnosis and 3.5% of relapse samples; *MYC* amplifications have been suggested to be targetable by aurora kinase inhibitors, with various trials ongoing [31]. Alterations were also found in *BRCA1/2*, *ATM* and *MLH1*, and the third most common alteration in relapse samples was in *ARID1A*, which is associated with response to PARP and EZH2 inhibitors [81]. Alterations were also found in common NSCLC genes, such as *EGFR*, *KRAS* and *MET*. All *EGFR* mutated samples were “relapse samples” and it was proposed that these were obtained from SCLC-transformation patients who had previously had anti-EGFR therapy or were the result of histological misclassification [80]. This highlights a limitation of this study, which is that the samples were obtained retrospectively and with no independent verification of their histological status or treatment history.

Research in Manchester has also looked at the potential for ctDNA to determine therapeutic targets. In this analysis of 39 limited stage and 30 extensive stage SCLC patients, besides *TP53* and *RB1* alterations, *COL22A1*, *KMT2D* and *NOTCH1* were found to be commonly mutated [82]. In addition, 85% of patients had a mutation in one of 18 DNA damage repair genes. By comparing genomic changes with a database of targeted therapy options, cancer drivers were detected in 69% of samples, and 60% were linked to potential therapies. As in Almodovar’s study, serial sampling of *TP53* variant allele frequency (VAF) showed the majority of patients on platinum-based therapy had a reduction in VAF during treatment, before a rise post-treatment corresponding to radiological progression. Forty-eight patients had both ctDNA and CTC analysis, with 37 of the 48 having at least one CTC detected via CellSearch^®^ and 46 of the 48 having detectable ctDNA. The combination of CTC and ctDNA analysis is of interest, and while it was not performed in this study, it has been demonstrated that collection of CTCs and ctDNA can be performed together using the same blood sample bottle, and this may be of relevance to future clinical utility [83]. *TP53* VAF in ctDNA, high VAF and number of CTCs were all significantly related to poor survival [82].

Monitoring of SCLC using ctDNA has also been performed specifically in limited stage disease patients treated with curative-intent therapy. In one such study, 23 limited stage patients had serial sampling and of these, two had undergone surgical resection and the remainder had completed chemoradiotherapy. Follow up showed that detection of ctDNA subsequent to definitive treatment was associated with increased risk of disease relapse, with poorer PFS and OS [84]. Indeed, the PFS difference was striking, with patients who had never had ctDNA detected post-therapy having a median PFS of 48 months, compared to 9.1 months in those who did have detectable ctDNA. The clinical benefit of selecting patients who are at higher risk of relapse following curative therapy is clear and has significant scope to improve patient experience and survival. The same group has also shown that ctDNA VAF is related to tumour volume, providing another potential use as a biomarker in SCLC [85].

ctDNA can additionally be used to obtain genomic information aside from common alterations such as mutations and amplifications. Indeed, given the only meaningful success in SCLC therapy in the last decade has come from the addition of immunotherapy to first-line therapy, there was interest in determining if blood-TMB (bTMB) from ctDNA could be a more useful biomarker than the tissue equivalent. Unfortunately, recent evidence appears to show this is not the case [86]. Epigenetic changes can also be obtained from ctDNA, with methylated *SHOX2* (*mSHOX2*) in plasma being shown to correlate to response in both NSCLC and SCLC during systemic treatment [87]. Blood-based epigenetic changes have further been investigated for a role in subtyping and diagnosing lung cancers [88]. Similarly, another liquid biopsy method, microRNAs (miRNAs), have been shown to also be valuable in the diagnosis and subtyping of lung cancers with high diagnostic accuracy [89].

Taken together, the above demonstrate that ctDNA is certainly a key candidate for moving liquid biopsy into the clinical environment, and interest in its potential role is only increasing with time. However, as with CTC analysis, the current evidence-base would benefit from prospective trials to confirm the clinical utility of this technology.

## 4. Future Role of Liquid Biopsy in Clinical Care

Evidence has accumulated in the past decade to show a potential role for liquid biopsy, particularly in the form of CTC and ctDNA analysis, in the clinical care of patients with SCLC for various indications such as treatment selection, monitoring, prognosis and prediction of treatment benefit. However, despite validated methods, the clinical utility of these approaches has not been proven outside of a clinical trial setting and the immediate application of liquid biopsy in the absence of any genomically targeted therapy is remote.

Although there is a body of evidence to show that the number of CTCs per 7.5 mL of blood prior to treatment, after one cycle of treatment and also reduction in CTC number between samples corresponds closely to prognosis in patients, the utility for treatment-based decisions is limited by the lack of a standard prognostic cut-off and by the nature of CTCs being a continuous rather than dichotomous variable. The question remains whether patients with a worse or better prognosis according to CTC number would benefit from an alternative treatment, especially as patients have highly variable priorities in terms of additional survival benefits they would consider meaningful, and the extent of adverse events they are willing to accept. With SCLC being rapidly progressive, and with numerous symptomatic effects from tumour bulk such as superior vena cava obstruction, chemotherapy can have significant benefits to patient wellbeing. However, this must be balanced against the known risks of multi-agent or even single-agent chemotherapy, which can include serious outcomes such as neutropenic sepsis [2,10].

There is clearly a subset of patients who have highly chemorefractory disease, and in these cases, chemotherapy is likely to cause overall harm with little benefit and impede the quality of life for these patients. Indeed, it is known that mortality within 30 days of chemotherapy administration is a concern in SCLC [10]. CTCs, with their clear prognostic role and emerging role in chemosensitivity, would therefore be an ideal candidate biomarker to select patients for whom chemotherapy should not be attempted, or at least in whom a clear informed consent process should be undertaken with the patient and their family. So far, a link between 30-day mortality post-chemotherapy and baseline CTC level has yet to be clearly demonstrated, but this is an ideal area for future research. Conversely, patients with extremely low ctDNA or CTC levels, providing other investigations are acceptable, could potentially be “re-staged” for consideration of more localised treatments such as surgery or radiotherapy [56,57]. Given the difficulties in improving systemic therapy in SCLC in recent decades, greater stratification to these other treatment modalities may assist in improving overall SCLC survival.

Arguably the greatest potential for CTCs currently is their use in preclinical studies to better understand SCLC biology, chemoresistance and phenotypic subtypes. SCLC has been recognised in the United States as a “recalcitrant cancer” as per the Recalcitrant Cancer Research Act of 2012, which directs the national cancer institute (NCI) to develop research frameworks for cancers with a five-year survival below 20% and estimated to cause at least 30,000 deaths per year in the USA [90]. Key recommendations from the NCI’s Scientific Framework for Small Cell Lung Cancer report included the development of novel models for the study of all the phases of SCLC and comprehensive genomic profiling studies in SCLC to improve knowledge of the molecular abnormalities that exist both at diagnosis and following therapeutic relapse [91]. CTCs, as a proxy for main tumour bulk, can offer an excellent resource for elucidating the molecular and biological methods employed by the disease for metastatic spread and chemoresistance preclinically. However, the lack of availability of the complex technology to assess CTCs in routine clinical laboratories presents a major barrier to the routine use of CTCs to guide current therapy decisions. Within the clinical research setting, there is an argument to stratify patients in clinical trials according to CTC number, particularly in earlier phase trials with relatively small numbers of patients and in trials where CTCs can be obtained for reverse translation in preclinical models [58,63]. For the time being, these research-based indications are most likely to be where the clinical utility of CTCs is greatest.

ctDNA, potentially sampled at routine blood draws and being easily transported in storage containers or as frozen plasma, offer a significant advantage over the complex analysis of CTCs. While CTC enumeration may therefore be limited to large academic centres and clinical trials, ctDNA has much broader potential clinical utility. This is only likely to accelerate as genomic technology continues to advance and such analysis becomes ever-more cost-effective. This may be why, in contrast to CTCs, ctDNA is increasingly employed as a routine diagnostic in patients with NSCLC, with technology being adopted widely in clinical laboratories [92]. While ctDNA analysis in NSCLC is mainly focused on treatment selection, given the significant issue of chemorefractory disease in SCLC, serial ctDNA sampling after the first cycle of chemotherapy could be used to determine if the current treatment strategy should be continued. If there is evidence that the disease is not responding, it could provide an indication to switch to an alternative option (which may be further systemic therapy, radiotherapy or supportive care). Liquid biopsy in this case would have unique benefits compared to standard radiological monitoring. However, such a protocol in practice would ideally benefit from a larger menu of therapeutic modalities in SCLC to use as an alternative to the failing therapy.

It is generally accepted that for a DNA-based investigation to be ready for use in the clinic it is required have evidence of analytical validation, clinical validation, clinical utility and meet ethical and legal standards [93]. While numerous studies have shown the analytical and clinical validation of ctDNA in SCLC, and clinical utility has been demonstrated from ctDNA in NSCLC [94], clinical utility is currently lacking for this investigation in SCLC. To confirm the clinical utility of ctDNA as a biomarker in SCLC, well designed prospective clinical trials are required. For example, use of ctDNA as a “mid-treatment line” liquid biopsy to change treatment strategy (or following initial chemoradiation in the context of limited stage disease) would need trials comparing this change in management based on ctDNA, against the current gold standard. A demonstrated improvement would be required in either survival, or in patient reported outcomes such as quality of life. Such trials are essential to confirm the clinical utility of ctDNA as biomarker-investigation in SCLC, and would be of particular benefit in patients with difficult to interpret radiology [95].

While biomarker-directed therapy for SCLC has yet to enter the clinic, there is cautious optimism that ongoing efforts to molecularly subtype SCLC in tissue, combined with the emerging role of liquid biopsy methods [14,80], will reveal distinct subsets which can be exploited to improve therapy using existing or next-generation treatments [96,97]. While advantages and disadvantages are inherent in these liquid-based technologies, they offer complimentary methods that are less invasive, can be obtained serially, and offer the possibility to track and dissect the evolutionary biology of the disease. The recent success of the addition of immunotherapy into first-line treatment shown by the CASPIAN and IMPOWER133 studies has put particular emphasis on selecting patients who would benefit most from this treatment [8]. Unfortunately, bTMB has not been shown to be prognostic, in contrast to in other cancer types [86], but it is clear that liquid biopsy has a unique potential to provide a well-needed immunotherapy biomarker in SCLC.

In summary, both CTCs and ctDNA have shown great promise as a method to improve the clinical care of small cell lung cancer patients. Both technologies have distinctive benefits and disadvantages, and perhaps the most powerful use of these technologies would be in tandem. A concerted effort to employ rigorous biomarker analysis using these tools will surely accelerate progress in understanding this deadly disease and identify new insights for translation to the clinic. Given the analytical issues in CTCs, the clinical role of this technology may be limited to large academic centres and clinical trials. However, as ctDNA and wider genomic testing becomes routinely available, ctDNA has broad potential to improve SCLC clinical care, providing that well designed, prospective clinical trials can prove that such a revolution would be of benefit to patients.

## Figures and Tables

**Table 1:** Summary of current issues in SCLC which may be addressed by the use of liquid biopsy methods, and the current state of the evidence base for their use.

Current Issue in SCLC	Potential Role of Liquid Biopsy	Strength of Evidence
Paucity of SCLC tumour tissue	Use of liquid biopsy to obtain sufficient tumour material for biomarker and genetic analysis	Significant evidence of high concordance between CTC/ctDNA and tissue biopsy samples
Lack of biopsy material after initial diagnosis	Ease of serial liquid biopsy permits re-assessment during and after treatment	Evidence for feasibility and clinically relevant turnaround times in ctDNA
Lack of established biomarkers in SCLC	Increased molecular and genomic analysis options to derive biomarkers	Clinical utility not fully established
Mortality within 30 days of chemotherapy administration	Select for those most at risk of 30-day mortality post-chemotherapy, or results within the cycle to abort futile therapy	Further studies required to confirm utility
Difficulty in determining effectiveness early in treatment	Liquid biopsy within first cycle to determine likelihood of response and permit change in therapy before clinical deterioration	Prospective trials needed to confirm clinical utility
Limited advancements in effective treatment options or molecularly targeted agents, especially beyond the second-line	Generation of patient derived explants from CTCs for use in preclinical research, ctDNA to evaluate efficacy within clinical trials	Research use only

**Table 2:** Key findings from studies analysing the prognostic effect of CTCs taken at baseline, after one cycle or completion of treatment, and change in CTC level.

Paper	Stage of Disease	Threshold Used	Baseline CTC	CTC after One Cycle	CTC Post Treatment	Reduction in CTCs
Hou 2012 [49]	Limited and Extensive	50	PFS 4.6 vs. 8.8 m (*p* < 0.001) OS 5.4 vs. 11.5 m (*p* < 0.001)	PFS 4.1 vs. 9.6 m (*p* = 0.001) OS 4.1 vs. 10.4 m (*p* < 0.001)	Not available.	Conversion from <50 CTC at baseline to ≥50 after one cycle was associated with worse prognosis.
Naito 2012 [48]	Limited and Extensive	8	OS 8.5 vs. 17.2 m (*p* = 0.0014)	Not available.	Post-treatment survival 4.1 vs. 13.9 m (*p* = 0.0096)	Median survival time 7.2 vs. 4.1 m for those ≥8 to <8 compared to those which stayed ≥8.
Hiltermann 2012 [50]	Limited and Extensive	Lowest and highest quartile (2, 215)	Median survival 157 vs. 729 days (highest to lowest quartiles)	PFS 2.9 vs. 10.7 m (*p* < 0.001) OS 8.1 vs. 12.3 m (*p* < 0.001)	PFS 3.9 vs. 7.9 m (*p* = 0.007) OS 8.1 vs. 12.3 m (*p* = 0.05)	Not available.
Normanno 2014 [55]	Extensive	Not available.	Not available.	Not available.	Not available.	Median OS 4.2 vs. 7.2 m for CTC change ≥−89% and <−89% (*p* = 0.009).
Wang 2017 [59]	Limited and Extensive	2	PFS 6.1 vs. 10.7 m (*p* = 0.008)	PFS 4.9 vs. 10.5 m (*p* < 0.001)	Not available.	Improved PFS when CTC remained <2 (12.5 m) vs. <2 at baseline and ≥2 after one cycle (6.3 m) or ≥2 at both (4.7 m).
Aggarwal 2017 [53]	Limited and Extensive	5	PFS 6.7 vs. 11 m (*p* = 0.0259) OS 12.9 vs. 15.5 m, no relationship	PFS 3.2 vs. 10 m (*p* < 0.001) OS 9.0 vs. 18 m (*p* = 0.0001)	Not available.	Consistent decline in CTCs during chemotherapy lead to longer PFS but no statistically significant difference.
		50	PFS 4.8 vs. 10 m (*p* = 0.0002) OS 11.8 vs. 20.2 m (*p* = 0.0116)	PFS 2.6 vs. 9.6 m (*p* < 0.0001) OS 6.1 vs. 17.6 m (*p* = 0.0002)		
Tay 2019 [56]	Limited	5, 15, 50 (15 optimal)	PFS 5.5 vs. 19 m (*p* < 0.001) OS 5.9 vs. 26.7 m (*p* < 0.001)	Not available.	Not available.	Not available.

m: Months, OS: Overall Survival, PFS: Progression-Free Survival, CTC: Circulating Tumour Cell. Survival values rounded to one decimal place.
